# A clinical approach to new-onset psychosis associated with immune dysregulation: the concept of autoimmune psychosis

**DOI:** 10.1186/s12974-018-1067-y

**Published:** 2018-02-13

**Authors:** Souhel Najjar, Johann Steiner, Amanda Najjar, Karl Bechter

**Affiliations:** 1 Department of Neurology, Zucker School of Medicine at Hofstra/Northwell, 8 Black Hall, 130 E 77th Street, New York, NY 10075 USA; 2 Department of Psychiatry, University of Magdeburg, Leipziger Str. 44, D-39120 Magdeburg, Germany; 3 Department of Neurology, Lenox Hill Hospital, 8 Black Hall, 130 E 77th Street, New York, NY 10075 USA; 4 Ulm University, Ludwig-Heilmeyer-Str. 4, D-89312 Günzburg, Germany

**Keywords:** Autoimmune, Psychosis, NMDAR, Encephalitis, Neuroinflammation, Microglia, Neuronal, Synaptic, Antibodies, Neuroimaging

## Abstract

Growing data point to the overlap between psychosis and pathological processes associated with immunological dysregulation as well as inflammation. Notably, the recent discovery of antibodies against synaptic and neuronal cell membrane proteins such as anti-*N*-methyl-d-aspartate receptor provides more direct evidence of the etiological connection between autoimmunity and subsequent hazard of psychosis. Here, we advocate the use of term “autoimmune psychosis,” as this term suggests that autoimmune disorders can masquerade as drug-resistant primary psychosis, and this subtype of psychosis has anatomical and immunological footprints in the brain, despite the frequent absence of structural abnormalities on conventional brain MRI. Furthermore, this term might serve as a reminder not to overlook appropriate neurological workup such as neuroimaging and EEG testing, as well as CSF analysis, for cases with acute or subacute atypical onset of neuropsychiatric presentations including those dominated by acute psychotic symptoms. We propose etiologically and serologically oriented subclassification as well as multi-modal diagnostic approach to address some of the challenges inherent to early diagnosis of patients presenting with atypical and refractory new-onset psychotic symptoms of autoimmune origin. This is particularly relevant to the diagnosis of seronegative but probable autoimmune psychosis (SPAP) that might masquerade as antipsychotic drug-resistant primary psychotic disorder. This distinction is therapeutically important as autoimmune-related psychotic symptomatology can frequently respond well to timely treatment with proper immune modulatory therapies.

## Background

Human and animal data suggest that neuroinflammatory and immunological abnormalities can contribute to the neurobiology of primary psychotic disorders [1, 2]. Human post-mortem brain, serological, cerebrospinal fluid (CSF), neuroimaging, genetic, and epidemiological studies have documented diverse inflammatory and immunological abnormalities in a subgroup of patients diagnosed with schizophrenia according to the currently established DSM/ICD criteria [1–4]. Among the main inflammatory abnormalities are microglial activation and proliferation in brain regions of functional relevance to psychosis (e.g., dorsolateral prefrontal, superior temporal, and anterior cingulate cortices), and upregulation of inflammatory mediators such as pro-inflammatory cytokines and matrix metalloproteinases [1–4]. Findings of post-mortem studies have not only linked microglial activation to the neurobiology of psychosis but also to suicidal state [3, 4]. Recent meta-analysis of post-mortem brains documented statistically significant increase in microglial density and pro-inflammatory gene expression, without significant alteration in the density of other glial cells or anti-inflammatory gene expression in schizophrenia compared with healthy subjects [5]. Studies documenting elevated serum levels of beta2-microglobulin in drug-naïve schizophrenia compared with healthy cohorts [6], with further elevation of its levels following treatment with antipsychotic medications, [7] suggest that immune dysregulation may play a role in a subgroup of these patients. Various CSF immunological and inflammatory changes are also well documented in a subset of individuals with drug-resistant schizophrenia [8, 9]. These changes include increased “CSF: serum albumin ratio” indicative of blood-CSF barrier hyperpermeability; increased intrathecal synthesis of IgG, IgM, and/or IgA; and presence of up to four IgG oligoclonal bands, mild pleocytosis, elevated neopterin levels, and elevated cytokines [8, 9]. These observations, together with documented elevated levels of S100B protein, a marker of glial activation and blood-brain barrier hyperpermeability, in the brain, CSF as well as blood support the hypothesis that hyperpermeability and/or disruption of blood-brain and blood-CSF barriers may contribute to the neurobiology of psychosis, in part via transendothelial inflammatory cell migration and increased cross-talk between brain innate immunity and peripheral adaptive immunity [1, 2, 8–10]. This is also supported by the post-mortem ultrastructural findings of inflammatory and degenerative changes affecting neurovascular unit in schizophrenia [1, 2].

In addition, several epidemiological studies reported a strong bidirectional relationship between autoimmune disorders and psychosis [2, 11]. One study showed a positive correlation between brain-reactive antibodies associated with autoimmune disorders and subsequent hazard of psychosis [11], although the abundance of brain-reactive IgG antibodies in the cortex of individuals with schizophrenia was shown in another study to be similar to that of healthy controls [12].

Moreover, the strong association between both early infections and increased risk of developing psychosis in the adult life [1, 11], as well as the significant increase in serum titers of anti-CMV and anti-HSV1 and anti-HSV2 IgG antibodies in schizophrenia patients [13], provide indirect argument in support of immune involvement [11]. Collectively, the above data point to the overlap between psychosis and pathological processes associated with immunological dysregulation as well as inflammation.

## Antibodies against neurotransmitter receptors and synaptic proteins in schizophrenia and new-onset psychosis: the concept of autoimmune psychosis

Early identification of antibodies targeting neurotransmitter receptors, such as 5-hydroxytryptamine 1A receptor and muscarinic cholinergic receptor 1, as well as dopamine-2 receptor, in individuals with schizophrenia suggests that autoimmunity plays a role in a subgroup of these patients [14–16]. Notably, the recent discovery of antibodies against synaptic and neuronal cell membrane proteins such as anti-*N*-methyl-d-aspartate receptor (NMDAR) and gamma-aminobutyric acid beta receptor (GABA_ß_R) provides more direct evidence etiologically linking autoimmunity-related dysregulation of glutamatergic and GABAergic neurotransmissions to subsequent hazard of psychosis [17–23]. One meta-analysis showed that patient with psychiatric illnesses such as schizophrenia and schizoaffective disorders are about three times more likely to have NMDAR antibodies (mostly of IgA or IgM type against GluN1 (NR1) subunit), compared with healthy controls [24]. Similarly, other studies reported higher, albeit variable, prevalence of IgG antibodies targeting GluN1 subunit of NMDAR in the sera of individuals with first-episode psychosis (ranging from 0 to 12%) [17–21, 25]. Furthermore, NMDAR antibody immunofluorescence response in schizophrenia patients with past catatonia is shown to be greater than that in healthy controls [26]. Additional studies revealed higher prevalence of other neuronal antibodies such as voltage-gated potassium channel (VGKC)- complex antibodies in patients with schizophrenia [27, 28]. On the contrary, the findings from other studies provide counterargument to the putative pathogenic significance of synaptic and neuronal antibodies in a subset of individuals with schizophrenia and new-onset psychosis. Several studies showed comparable prevalence of antibodies targeting synaptic and neuronal cell membrane protein among healthy and schizophrenia cohorts [20, 29]. Another recent study showed that the prevalence of these antibodies among individuals with first-episode psychosis was low and comparable to that of healthy controls [30]. The mixed results and the variability in the estimates of synaptic and neuronal antibody seropositivity may in part be attributed to methodological heterogeneity (e.g., cell-based assay (CBA) using live as opposed to fixed cells) that might have influenced the sensitivity and specificity of immunostaining assays used across these studies [24, 25].

The growing human and experimental data supporting an association between autoimmunity and risk of developing psychosis have prompted some authors to use the term “autoimmune psychosis” [17]. We advocate using the term “autoimmune psychosis” for those autoimmune disorders presenting primarily with atypical psychotic features masquerading as drug-resistant primary psychosis. This term suggests that this subtype of psychosis has anatomical and immunological footprints in the brain. It also serves as a reminder not to overlook appropriate neurological workup, (such as neuroimaging, EEG testing, and CSF investigation), but rather to apply it more broadly to formulate accurate differential diagnostic considerations that entertain underlying autoimmune process among other organic etiologies. Examples of atypical psychotic presentations suggestive of organic causes [31, 32] are (1) atypical age of onset; (2) predominance of particular symptoms such as confusion, disorientation, and language disintegration; (3) catatonia, given its diverse etiology; (4) predominance of visual or multi-modal hallucinations (visual and tactile); (5) olfactory hallucinations suggestive of mesial temporal lobe pathology; (6) predominance of specific delusions such as those related to misidentifications (Capgras syndrome); (7) antecedent or concurrent medical illness or systemic manifestations including significant weight loss; and (8) lack of predisposing risk factors for primary psychosis such as a strong family history of schizophrenia, a premorbid schizoid personality, or precipitating stress for mental disorder.

## Multi-modal diagnostic approach

We recommend multi-modal diagnostic approach to psychosis with suspected organic etiology, including immunological dysregulation, that greatly emphasize the importance of thorough clinical assessment before considering the ever-expanding options for costly ancillary diagnostic testing, with the main objective of augmenting timely recognition and treatment of this unique subgroup of psychosis. The clinical evaluation includes obtaining detailed medical and neurological history, performing thorough general and neurological examinations, recognizing atypical psychotic presentations, and identifying clinical accompaniments suggestive of immune dysregulation after excluding other organic causes [17, 33, 34].

The clinical accompaniments [17, 33, 34] are (1) atypical psychotic presentation [31, 32]; (2) neurological disturbances such as rapidly evolving encephalopathy, seizures including faciobrachial dystonic seizures pathognomonic for limbic encephalitis associated with leucine-rich-glioma inactivated 1 (LGI1) antibody, abnormal movements, autonomic instability, language disintegration, and reduced level of consciousness; (3) antecedent systemic symptoms or viral-like febrile illness suggestive of prodromal phase of autoimmunity; (4) unexplained soft or overt findings on neurological examination; and (5) strong personal or family history of autoimmune disorders.

The ancillary diagnostic abnormalities suggestive of underlying neuroimmune dysregulation [32–38] include the followings: (1) elevated serum levels of antibodies targeting synaptic and neuronal cell membrane proteins, or biomarker characteristic for comorbid autoimmune disorders such as systemic lupus erythematous and Hashimoto’s encephalopathy among many others; (2) CSF, neuroimaging, and EEG abnormalities after excluding other organic causes such as infectious etiologies: (a) CSF abnormalities include antibodies targeting synaptic and neuronal cell membrane proteins, lymphocytic pleocytosis, oligoclonal bands, elevated IgG index, and elevated neopterin level (a non-specific marker of T helper cell 1 activation-dependent immune response) [35]; (b) neuroimaging structural and functional abnormalities such as unilateral or bilateral hippocampal MRI FLAIR/T2 signal hyperintensities suggestive of autoimmune limbic encephalitis [33], as well as hyper-, hypo-, or heterogeneous cerebral glucose metabolism on FDG- positron emission tomography (PET) or altered cerebral blood flow on single photon emission computed tomography (SPECT) [33, 36, 37]; we suspect that FDG hypermetabolism, as opposed to hypometabolism, might be more indicative of inflammation after excluding alternative causes [38]; and (c) EEG abnormalities such as epileptiform discharges, delta brushes [33].

## Autoimmune disorders presenting with new-onset psychotic symptoms

We propose that psychotic presentations secondary to immune dysfunction may be categorized into three groups according to their specific etiology and serum as well as CSF immunological profiles (Table 1). This subclassification might facilitate early consideration of these subgroups of autoimmune disorders in individuals presenting primarily with atypical new-onset psychosis of uncertain organic causes masquerading as refractory primary psychosis.

1. Psychosis associated with autoantibodies targeting well-characterized synaptic and neuronal cell surface proteins [3–5]. Indeed, findings from several case reports and clinical case series have linked autoantibodies targeting NMDAR, α-amino-3-hydroxy-5-methyl-4-isoxazolepropionic acid receptor (AMPAR), GABA_ß_R and VGKC-protein complex, to new-onset psychosis among other psychiatric disturbances (e.g., mania, anxiety, aggression, emotional lability, and personality changes) as the dominant presenting symptoms [17–23, 27, 38–42]. Although the majority of these patients eventually exhibit a wide constellation of neurological symptoms throughout the course of the illness, isolated neuropsychiatric symptoms with dominant psychosis may rarely remain the key symptoms; we suggest that the latter pattern might constitute the presentation of milder form or forme fruste of autoimmune encephalitis. Thus, we recommend screening for these antibodies in serum and CSF of individuals presenting with new-onset atypical drug-refractory neuropsychiatric symptoms including new-onset psychosis, particularly in those exhibiting any of the above highlighted clinical accompaniments. This is important, as structural neuroimaging abnormalities are commonly not noted on conventional MRI in patients with autoimmune encephalitis associated with these synaptic and neuronal surface autoantibodies [43, 44]. Indeed, recent studies of anti-NMDAR encephalitis showed that structural neuroimaging abnormalities are absent on initial presentation and follow-up brain MRIs in about 89 and 79% of cases, respectively, despite associated profound cognitive, neurological, and neuropsychiatric disturbances [43]. Furthermore, identification of specific autoantibodies does not only establish the diagnosis of autoimmune psychosis, but also can clarify its subtype and guide treatment.

2. Psychosis associated with classical systemic inflammatory and autoimmune disorders (e.g., neuropsychiatric systemic lupus erythematosus), neuroinflammatory disorders (e.g., inflammatory demyelinating disorders such as multiple sclerosis and acute disseminated encephalomyelitis, and neurosarcoidosis), Hashimoto’s encephalopathy, or autoantibodies targeting intracellular antigens (i.e., glutamic acid decarboxylase and onconeuronal antigens).
  Neuropsychiatric systemic lupus erythematous (NPSLE) occurs in 25 to 75% of SLE patients [1]. NPSLE includes cognitive deficit, headache, psychiatric symptoms (e.g., anxiety, depression, and psychosis), seizures, movement disorders, non-specific demyelination, and cerebrovascular events [32, 45, 46]. Psychiatric symptoms often occur within the first 2 years of disease onset [1] and are rarely limited to isolated psychosis [32]. The pathophysiology of NPSLE is multifactorial and includes inflammation-mediated microangiopathy and autoantibodies such as anti-GluN2 (NR2) subunits of NMDAR, anti-ribosomal P (positive in 90% of psychotic SLE patients), anti-endothelial cell, anti-ganglioside, and anti-dsDNA, as well as anti-phospholipid antibodies [1]. Other systemic inflammatory disorders such as Sjögren’s syndrome, vasculitis, and Behçet’s disease can also present with a variety of neuropsychiatric features including psychosis, in addition to a wide constellation of neurological disturbances such as cognitive changes [18]. Please refer to the comprehensive review published on this topic [18].
  Central nervous system (CNS) inflammatory demyelinating disorders such as acute disseminated encephalomyelitis [47] and multiple sclerosis [48] can present with diverse psychotic features, suggesting that these disorders may share some common immune and inflammatory mechanisms with primary psychotic disorders [49]. Neurosarcoidosis is another neuroinflammatory disorder that can have neuropsychiatric comorbidities with psychotic features. Indeed, psychotic manifestations can be seen in up to 17% of individuals with neurosarcoidosis [50].
  Hashimoto’s encephalopathy is a relatively uncommon steroid-responsive encephalopathy. It is characterized by rapidly progressive non-specific encephalopathy, seizures, myoclonus, stroke-like episodes, and diverse neuropsychiatric features that include mood disorders as well as psychosis [33]. This disorder is associated with thyroid antibodies, which can are also present in about 13% of healthy individuals [33]. Brain MRI and CSF investigations may be normal or revealing non-specific abnormalities [33].
  Glutamic acid decarboxylase antibody seropositivity has been associated with a variety of neurological disorders including limbic encephalitis, which typically present with neurological (e.g., cognitive, short-term memory impairment, confusion, seizures) and psychiatric symptoms (mood alteration, anxiety, psychosis) [1, 33]. However, studies that are more recent indicated that other concomitant antibodies, such as those targeting GABA_ß_R, are more likely to be the culprit [51, 52].
  Paraneoplastic limbic systems can also present with a variety of neuropsychiatric including psychosis and neurological symptoms such as seizures and cognitive impairment [33]. Although onconeuronal antibodies are associated with paraneoplastic CNS syndromes, direct cytotoxic effects of T cell immunity appear to be more etiologically relevant to pathophysiology of paraneoplastic limbic encephalitis [53].

3. Seronegative but probable autoimmune psychosis (SPAP). The evidence supporting the relevance of SPAP as a distinct diagnostic category in clinical practice is indirect, limited, and largely consists of case reports that document responsiveness of secondary psychosis to immune therapies despite negative comprehensive serological assays for systemic and central nervous system autoimmune diseases, as well as lack of any other discernable organic etiology [54, 55]. The molecular and cellular changes of the pathoimmunological processes underlying SPAP remain largely mysterious. However, some of these cases may represent a subgroup of the increasingly recognized spectrum of seronegative autoimmune encephalitides [33, 50, 54–61] presenting primarily with acute or subacute onset encephalopathy, short-term memory impairment, and neuropsychiatric features with dominant psychotic symptoms [54, 55]. Its etiological connection to the underlying immunological dysregulation can be inferred only after excluding other alternative organic causes such as infectious, toxic-metabolic, substance abuse, neurodegenerative, or genetic disorders. We suggest that seronegativity is likely attributed to autoantibodies that either have not yet been discovered or present exclusively in the CSF that is often not analyzed in this subgroup of individuals; (I) many cases of paraneoplastic limbic encephalitis previously characterized as seronegative were subsequently found to be GABA_ß_R antibody seropositive [52], and (II) NMDAR antibodies were detected only in CSF of about 13% of individuals with confirmed anti-NMDAR encephalitis through research-based combined testing of immunohistochemistry and CBAs [62]. The strong immunoprecipitation property of the brain can also result in high binding of autoantibodies to their respective receptors, thus limiting their free circulation and detection in the serum and CSF [63]. Furthermore, methodological heterogeneity might occasionally contribute to false seronegativity; although the current CBA testing using commercially available kits is very specific, it appears to be less sensitive for detecting antibodies targeting synaptic and neuronal cell surface antigens (e.g., NMDAR, GABA_ß_R, AMPAR) in patients with suspected synaptic autoimmune encephalitis, as compared to research-based combined testing of both rodent brain immunohistochemical staining as well as CBAs [64]. Indeed, about 12% of all confirmed autoimmune encephalitis cases were identified only after demonstrating positive rodent brain reactivity on immunohistochemical staining followed by research-based CBAs [64]. Alternatively, CNS autoimmunity may be mediated by not-yet-defined innate- or T cell autoimmunity, instead of circulating autoantibodies [1]. Thus, diagnosing SPAP requires high index of clinical suspicion together with lower threshold for referring patients with suspected SPAP for basic diagnostic investigation such as neuroimaging, EEG testing, and CSF analysis. However, given the relatively low diagnostic yield of conventional brain MRI [43, 44] and the limited use of CSF analysis, it is conceivable that SPAP frequently escapes clinical detection. Indeed, findings from recent studies assessing the sensitivity of various diagnostic tests in autoimmune encephalitis indicate that brain FDG-PET offers a higher diagnostic yield, with cerebral cortical hypometabolism being the most frequently observed abnormality, compared with early EEG, conventional brain MRI, and even CSF analysis [65]. A clinically meaningful response to immune therapy trial, similar to the approach used in seronegative rheumatologic disorders [66], might provide indirect evidence of the underlying CNS autoimmunity, particularly when associated with restoration of functional neuroimaging abnormalities of glucose metabolism or blood flow following immune therapy [36, 67, 68]. The yield of brain biopsy, similar to that of autoimmune encephalitis, is likely low [41]. However, brain biopsy might be considered in carefully selected cases of severe encephalopathy associated with refractory psychotic features of undefined organic causes to confirm their etiological connection to autoimmunity before initiating more aggressive immunosuppressive therapies. Indeed, we have reported cases and reviewed literature of brain biopsy-proven seronegative autoimmune encephalitis presenting with isolated neuropsychiatric disorders including psychosis [54, 56]. Although the typical neuroinflammatory findings of perivascular and parenchymal mononuclear inflammatory infiltrates and/or microglial nodules are typically not etiologically specific, but can, in the proper clinical context and after excluding other organic etiologies such as infections, provide indirect evidence of probable neuroimmune process.

**Table 1:** Etiological subclassification of autoimmune psychosis

Autoimmune Psychosis Subclass	Autoantibodies and proposed inflammatory mechanisms	Associated neurological and neuropsychiatric symptoms
Autoimmune encephalitis associated with synaptic and neuronal cell membrane proteins antibodies	NMDAR antibodies [IgG against NR1 (Glu1) subunit]	Cognitive decline, memory loss, speech fragmentation, seizures, dyskinesias, catatonia, autonomic instability, and central hypoventilation. Delta brush on EEG.
	AMPAR antibodies (IgG against GluR1/2 subunits)	Cognitive decline, memory loss, affective changes, confusion, agitation, delirium, and seizures
	GABA_ß_R antibodies (IgG against B1 subunit)	Seizures, memory loss, and confusion
	VGKC-protein complex antibodies: LGI1 antibodies	Amnesia, confusion, disorientation, autonomic dysfunction, apathy, irritability, faciobrachial dystonic seizures, and hyponatremia
	CASPR2 antibodies	Neuromyotonia, muscle spasm, fasciculation, neuropathic pain dysautonomia, confusion, amnesia, insomnia, and seizures
Psychosis associated with autoimmune and inflammatory disorders		
1. Classical systemic autoimmune disorders:		
Neuropsychiatric systemic lupus erythematosus	NMDAR antibodies [IgG against NR2 (Glu2) subunit] Ribosomal P antibodies	Cognitive changes, affective disorders, anxiety, delirium, seizures, and neurovascular disease
2. Neuroinflammatory disorders:		
Inflammatory demyelinating disorders (MS, ADEM)	OCB antibodies Anti-myelin antibodies: MBP-antibodies MOG-antibodies	Focal symptoms and neurological deficits, optic neuritis; characteristic CSF analysis and brain imaging findings
3. Hashimoto’s encephalopathy	Antithyroid antibodies: TPO- and Tg-antibodies Autoimmune vasculitis Immune complex deposition	Encephalopathy, memory loss, confusion, altered level of consciousness, seizures, stroke-like symptoms, and myoclonus
4. Autoimmune encephalitis associated with antibodies targeting intracellular antigens	Onconeural antibodies (HU, MA2, CRMP5, Amphyphisin	Cognitive decline, short-term memory impairment, affective disorders, anxiety, seizures, neuropathy, cerebellar ataxia, and cranial neuropathy
	GAD antibodies; often associated with GABA_ß_ antibodies	Cognitive decline, short-term memory impairment, affective disorders, anxiety, seizures, neuropathy, ataxia, weight loss, cerebellar ataxia, stiff person syndrome
Seronegative but probable autoimmune encephalitis (SPAP)	Unknown autoantibodies Innate- or T-cell autoimmunity?	Encephalopathy, cognitive decline, memory impairment, affective disorders, and seizures

Abbreviations: *NMDAR N*-methyl-d-aspartate receptor, *AMPAR* a-amino-3-hydroxy-5-methyl-4-isoxazolepropionic acid receptor, *GABAuR* gamma-aminobutyric acid beta receptor, *VGK.C* voltage-gated potassium channel, *LGI1* leucine-rich-glioma inactivated 1, *CASPR2* contactin-associated protein-like 2, *MS* multiple sclerosis, *ADEM* acute disseminated encephalomyelitis, *OCB* oligoclonal band, *MBP* myelin basic protein, *MOG* myelin oligodendrocyte glycoprotein, *TPO* thyroid peroxidase, *Tg* thyroid globulin, *GAD* glutamic acid decarboxylase

## Conclusions and future directions

We believe that the suggested etiologically and serologically oriented subclassification as well as the multi-modal diagnostic approach might address some of the challenges inherent to early diagnosis of patients presenting with atypical drug-resistant new-onset psychosis with suspected link to immune dysregulation. This is particularly relevant to the diagnosis of SPAP that might masquerade as antipsychotic drug-resistant primary psychotic disorder. This distinction is also therapeutically important as autoimmune-related psychotic symptomatology can frequently respond well to timely treatment with proper immune modulatory therapies. However, building broader consensus evidence-based guidelines highlighting cost-efficient diagnostic workup and management of patients presenting with unusual new-onset psychotic symptoms with suspected autoimmune origin such as SPAP are warranted. Large-sample randomized clinical trials investigating the prevalence of autoimmune psychosis among individuals with new-onset psychosis are needed. The trial designs need to incorporate more CSF studies and more advanced multi-modal neuroimaging such as resting-state functional brain MRI for elucidating potentially characteristic neural network functional connectivity alterations [44] as well as PET utilizing a novel translocator protein (TSPO) radioligand for imaging microglia activation-mediated neuroinflammation [69].

## Abbreviations

AMPAR: α-Amino-3-hydroxy-5-methyl-4-isoxazolepropionic acid receptor
CBA: Cell-based assay
CNS: Central nervous system
CSF: Cerebrospinal fluid
GABA_ß_R: Gamma-aminobutyric acid beta receptor
GAD: Glutamic acid decarboxylase
LGI1: Leucine-rich-glioma inactivated 1
NMDAR: *N*-Methyl-d-aspartate receptor
SPAP: Seronegative but probable autoimmune psychosis
TSPO: Translocator protein
VGKC-protein complex: Voltage-gated potassium channel
